# Bull's-Eye and Nontarget Skin Lesions of Lyme Disease: An Internet Survey of Identification of Erythema Migrans

**DOI:** 10.1155/2012/451727

**Published:** 2012-10-24

**Authors:** John N. Aucott, Lauren A. Crowder, Victoria Yedlin, Kathleen B. Kortte

**Affiliations:** ^1^Department of Medicine, Johns Hopkins University, 10755 Falls Road, Suite 200, Lutherville, MD 21093, USA; ^2^Division of Clinical Research, Lyme Disease Research Foundation, 10755 Falls Road, Suite 200, Lutherville, MD 21093, USA; ^3^Department of Physical Medicine and Rehabilitation, Johns Hopkins University School of Medicine, Phipps 174, 600 North Wolfe Street, Baltimore, MD 21287, USA

## Abstract

*Introduction*. Lyme disease is an emerging worldwide infectious disease with major foci of endemicity in North America and regions of temperate Eurasia. The erythema migrans rash associated with early infection is found in approximately 80% of patients and can have a range of appearances including the classic target bull's-eye lesion and nontarget appearing lesions. *Methods*. A survey was designed to assess the ability of the general public to distinguish various appearances of erythema migrans from non-Lyme rashes. Participants were solicited from individuals who visited an educational website about Lyme disease. *Results*. Of 3,104 people who accessed a rash identification survey, 72.7% of participants correctly identified the classic target erythema migrans commonly associated with Lyme disease. A mean of 20.5% of participants was able to correctly identify the four nonclassic erythema migrans. 24.2% of participants incorrectly identified a tick bite reaction in the skin as erythema migrans. *Conclusions*. Participants were most familiar with the classic target erythema migrans of Lyme disease but were unlikely to correctly identify the nonclassic erythema migrans. These results identify an opportunity for educational intervention to improve early recognition of Lyme disease and to increase the patient's appropriate use of medical services for early Lyme disease diagnosis.

## 1. Introduction

Lyme disease (LD), which is caused by the tick-borne spirochete *Borrelia burgdorferi*, is an important cause of infection in endemic regions of North America and Eurasia and has a strong seasonality with the majority of the cases occurring during the months of May through August. In North America, over 90% of cases are reported from the East Coast of the United States, although significant numbers of cases are also reported from the upper Midwest of the United States and certain areas of Canada and the West Coast. In Eurasia, cases have been reported from temperate regions such as Germany [1, 2], Norway [3], Finland [4], and the United Kingdom [5]. Erythema migrans (EM) is the most common manifestation of LD, with at least 80% of infected persons developing a variation of this skin lesion in the first weeks of infection [6]. EM is characterized by a round red patch gradually expanding over time, typically reaching at least 5 cm or greater in size [7, 8]. The localized rash appears three to thirty days after an infected tick bite and disappears naturally if left untreated over days to weeks. Though it has been documented that EM can have various manifestations [9], the classic “target” shaped EM is best known in the literature and most common on public health information and handouts [10]. In reality, a classic target EM manifests in only approximately 20% of patients with EM, with the majority of EM lacking the central clearing or ring-within-a-ring pattern [7, 9]. 

LD is an emerging infectious disease in many areas of the world including North America and temperate Eurasia. LD is the most commonly reported vector borne disease in the US and the 3rd most common reportable infectious disease in the Northeast and Mid-Atlantic United States [11]. As such, LD is a public health concern for many people, especially in periurban residential areas of the northeast and Mid-Atlantic. However, a review of the published literature on LD has documented both under- and overdiagnosis of the EM rash of LD [12, 13]. Additionally, a study showed that up to 72% of physicians surveyed are not able to correctly identify the EM accompanying LD [14] when shown both EM as well as other rashes common in an ambulatory population. Early diagnosis of LD is dependent on patients seeking evaluation, which depends on patients' ability to recognize rashes or skin lesions that have a high probability of being EM. However, no literature to date has been published on patients' ability to recognize EM. The current study is aimed at determining the ability of the general public accessing an LD website to accurately identify the EM rash. It is presumed that individuals who conclude that a rash is not EM will be less likely to pursue medical services for evaluation of LD. By discovering how accurately the public is in identifying EM lesions, we can then predict which lesions would lead the general public to pursue health care services. Additionally, by determining the identification pattern, educational interventions for the public can be targeted to increase knowledge and, therefore, hypothetically increase pursuance of appropriate clinical services. The hypothesis tested was that respondents will be able to correctly recognize the classic target EM, but not nonclassic EM. 

## 2. Methods

### 2.1. Instrument Development

In order to develop the LD rash survey, a review of the literature was conducted for articles on EM and the reports of misidentification of EM in the diagnosis of LD. In addition, expert opinions were solicited from published authorities in the field when choosing examples of both classic target and nonclassic EM [15]. Both EMs and non-Lyme lesions were included in the survey. A determination could then be made of which EMs would most likely alert a potential surveyor to a possibility of LD and which non-Lyme lesions might lead to the incorrect self-diagnosis of LD. 

EM photographs were selected based on professional experience of one of the authors (JA) in order to represent the widest variety of EM appearances that were identified from a review of the current literature. Presentation of EM has been reported most commonly as a homogenous red lesion, with central clearing or a target appearance occurring less frequently in a US population [9, 15–21]. Also commonly reported are secondary disseminated lesions [16, 19–23], occurring in 8–25% of cases [7, 9, 15, 17]. Less common, but consistently reported manifestations of EM include vesiculopustular lesions [9, 15, 17, 20–22, 24] and lesions that develop a blue-purple bruise-like appearance [15, 18].

**Figure 1:**
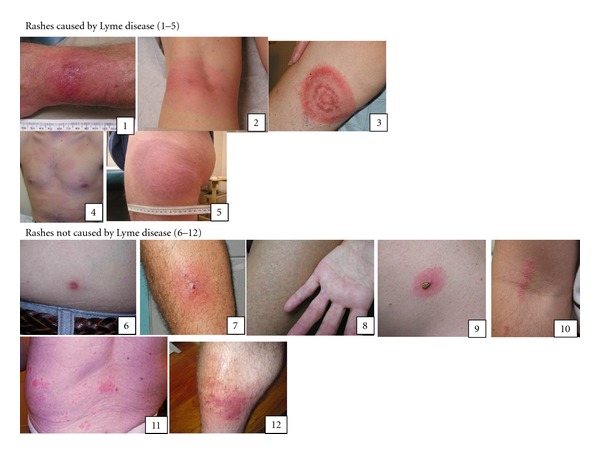
Rash photographs included in the website survey.

EM photographs included in the survey were a classic target EM as well as nonclassic EM: uniformly red lesion, disseminated cutaneous lesions, a vesiculopustular lesion, and a circular lesion with blue-purple coloration (Figure 1, numbers 1–5). We also included lesions commonly misdiagnosed as LD, as well as those lesions common in an ambulatory population [12, 25]. The rashes selected for this group included a small insect bite reaction, an immediate skin reaction with tick still attached, as well as rashes from poison ivy exposure, shingles, cellulitis, hand-foot-mouth disease, and *Staphylococcus aureus* infection (Figure 1, numbers 6–12). 

### 2.2. Administration

Access to the survey was available exclusively through the Lyme Disease Research Foundation website, which has been accessible to the public since 2007. Google search terms that directed users to the site include “Lyme disease,” “Lyme disease foundation,” “Lyme disease symptoms,” “symptoms of Lyme disease,” “what is Lyme disease,” “signs and symptoms of Lyme disease,” “treatment for Lyme disease,” and “signs of Lyme disease.” The website receives, on average, 10,000 hits per month, with peaks during the spring and summer seasons. Advertisement for the rash survey was accomplished through word-of-mouth, the Foundation's Twitter account, and a quarterly newsletter sent to those who signed up to receive it through the Foundation website. Once on the website, participants were directed via hyperlink to the survey, which was administered via http://www.SurveyMonkey.com/. The survey includes five rashes from known EM cases of early LD and seven rashes from common skin conditions not due to LD. Participants were asked to respond to one of the following three questions for each rash: “I am sure this is a Lyme disease rash,” “I am sure this is not a Lyme disease rash,” or “I do not know whether or not this is a Lyme rash.” After completion of the survey, regardless of performance, each participant was redirected to a detailed explanation of the causes of each rash. 

The survey was conducted between August 1, 2011 and January 31, 2012. SAS (Statistical Analysis System Institute, Cary, NC) and SPSS (IBM Corporation, Armonk, NY) were used for data analysis. This study was approved by the Johns Hopkins Institutional Review Board number NA_00071093.

## 3. Results and Discussion

### 3.1. Results

**Figure 2:**
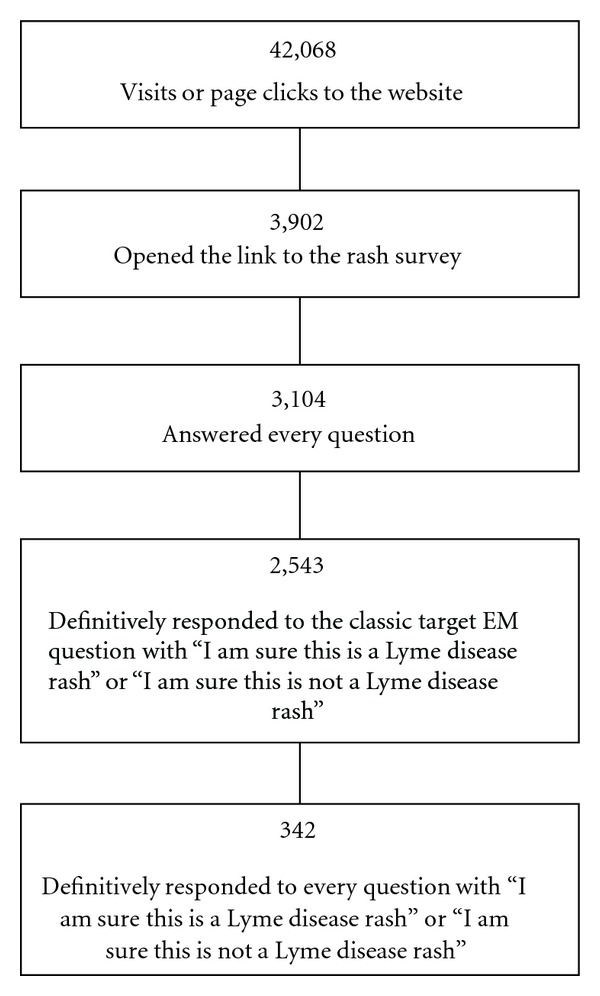
Number of responses at each stage of analysis.

**Figure 3:**
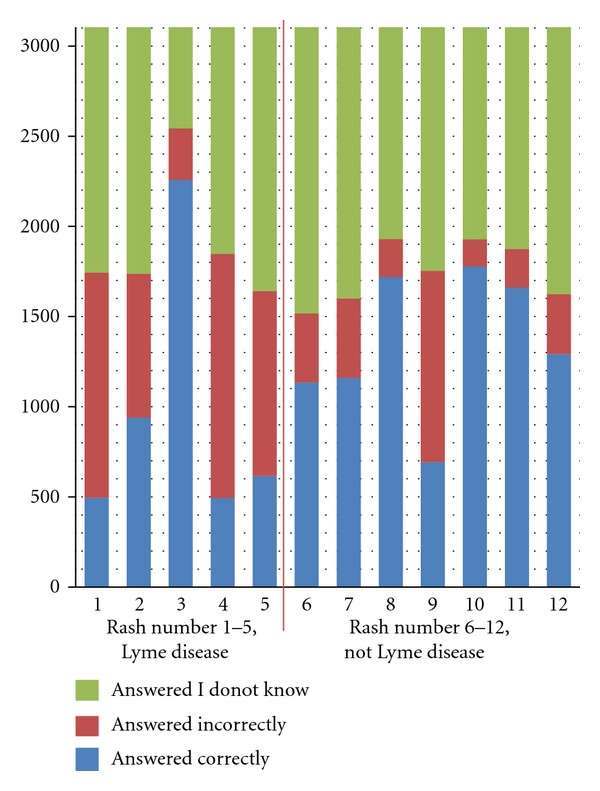
Responses to each rash of those who answered each question in the Lyme disease rash survey.

Over the six-month study period between August 1, 2011 and January 31, 2012, there were 42,068 visits or page clicks to the Lyme Disease Research Foundation website (Figure 2). Of the visitors to the website, 3,902 people were recruited by the rash survey and were redirected to the http://www.SurveyMonkey.com/ website where the survey was carried out. Of those visitors, 3,898 participated in the survey by answering at least one question, with 3,104 answering all questions.  Figure 3 shows the accuracy of the 3,104 respondents and shows the percentage of incorrect, correct, and unsure responses for each of the Lyme and non-Lyme rashes. Of the 3,104 participants who gave a response on all twelve rashes, 72.7% were able to correctly identify the classic target EM (Figure 1, number 3), 16.0% were able to identify the vesiculopustular EM (Figure 1, number 1), 30.3% were able to identify the uniformly red EM (Figure 1, number 2), 15.9% were able to identify the disseminated EM (Figure 1, number 4), and 19.8% were able to identify the blue-purple EM (Figure 1, number 5). Of those who gave a response on all twelve rashes, 9.2% incorrectly responded that the classic target EM was not due to LD, 40.2% incorrectly identified the vesiculopustular EM, 25.7% incorrectly identified the uniformly red EM, 43.6% incorrectly identified the disseminated EM, and 33.0% incorrectly identified the blue-purple EM. The survey included two non-Lyme rashes that are commonly misdiagnosed as being caused by LD. Of those who gave a response on all twelve rashes, 34.2% incorrectly identified the attached tick with an immediate skin reaction (Figure 1, number 9) and 10.7% incorrectly identified the rash from cellulitis (Figure 1, number 12) as being caused by LD. 12.3% incorrectly identified the small tick-bite reaction (Figure 1, number 6), 14.1% incorrectly identified the rash due to *Staphylococcus aureus* (Figure 1, number 7), 6.8% incorrectly identified the rash due to hand-foot-mouth disease (Figure 1, number 8), 4.8% were incorrect in identifying the poison ivy rash (Figure 1, number 10), and 6.9% incorrectly identified the rash due to shingles (Figure 1, number 11) as being caused by LD.

There was a high degree of uncertainty throughout the survey, regardless of lesion type. The classic target EM had an 18.1% uncertainty rate (i.e., percentage of participants who chose “I do not know whether or not this is a Lyme rash.”), the vesiculopustular EM had 43.8% uncertainty, the uniformly red EM had 44.0% uncertainty, the disseminated EM had 40.5% uncertainty, and the blue-purple EM had 47.1% uncertainty. There was also a high percent of uncertainty among those rashes not caused by LD. The small tick-bite reaction had 51.1% uncertainty, the rash due to *Staphylococcus aureus* had 48.5% uncertainty, the hand-foot-mouth rash had 37.8% uncertainty, the attached tick with an immediate skin reaction had 43.5% uncertainty, the rash due to poison ivy had 37.9% uncertainty, the shingles rash had 39.6% uncertainty, and the cellulitis rash had 47.6%. For those participants who correctly identified the classic target EM, they were able to better identify compared to all others (both incorrect and unsure) whether all other rashes were caused by LD or not (*χ* ^2^ values for all rashes >12.73, *P* < 0.003 for all relationships).

In order to exclude individuals who were unsure about the classic target EM and focus in on more definitive responders, we removed from analysis those individuals who indicated for the classic target lesion that they were unsure whether it was a rash due to LD or not, leaving *n* = 2543 for analysis (Figure 2). With this subset of our sample, those who correctly identified the classic target EM were better able to identify the disseminated EM (Figure 1, number 4) (*χ* ^2^ = 9.964, *P* = 0.007) and the blue-purple EM (Figure 1, number 5) (*χ* ^2^ = 37.942, *P* < 0.0001). However, they were also less able to correctly identify the attached tick with an immediate skin reaction photograph (Figure 1, number 9), incorrectly identifying it as LD when it was not as compared to participants who did not correctly identify the classic target EM as LD (*χ* ^2^ = 14.624, *P* = 0.001).

Finally, we excluded those participants who answered, “I am unsure whether or not this is a Lyme disease rash” on any question. This left 342 respondents who exclusively answered “I am sure this is Lyme disease” or “I am sure this is not a Lyme disease rash” for all questions (Figure 2). This was done because the levels of uncertainty in the initial population skewed the results. We followed the previously defined groups of those who were able to correctly identify the classic target EM and those who were not. This analysis showed that participants who correctly identified the classic target EM were better able to identify the disseminated lesions (*χ* ^2^ = 5.228, *P* = 0.022) and blue-purple rash (*χ* ^2^ = 5.978, *P* = 0.014) compared to those who were not able to correctly identify the classic target EM. However, this same group was less likely to be able to correctly identify the rash caused by hand-foot-mouth disease, inappropriately identifying it as caused by LD (*χ* ^2^ = 5.607, *P* = 0.018).

### 3.2. Discussion

Given that LD is the most common vector-borne disease in the United States and early treatment is associated with better outcomes, it is important that the general public have correct information about LD and how to identify the earliest signs of infection. Without knowledge of the most common sign associated with LD, the EM rash, it is unlikely that individuals will correctly identify the rash and seek out appropriate health care. The current survey study was aimed at determining whether a convenience sample of individuals accessing an LD website for information could accurately identify the classic and nonclassic rashes of LD versus rashes of other non-Lyme skin conditions. 

Overall, the findings support that among survey participants, the majority (73%) were able to correctly identify the classic target EM. However, approximately 1/5 of the sample was able to correctly identify the nonclassic appearances of EM suggesting that many people are unaware of the variations an EM can exhibit. The findings also suggest that non-LD lesions are misidentified as LD rashes, with the most common misidentification occurring with a picture of a localized reaction with the tick still attached to the individual. So there is a problem of both false negatives (i.e., concluding that a rash is not EM when in fact it is) and false positives (i.e., concluding that a rash is EM when in fact it is not). If the current sample represents the knowledge of the general population, then the majority of the population is unaware or has misinformation about the various presentations of EM. When there is a false negative error (i.e., concluding that a rash is not EM when in fact it is), then the individual may not seek out medical care as he/she erroneously concludes that he/she has a benign skin lesion such as a spider bite or bruise [26–28]. Additionally, because the EM resolves without antibiotic treatment in days to weeks [8], it may lead to the mistaken impression that the skin lesion was not due to an important disease. Without proper care in the early phases of the disease process, individuals are at much higher risk of recurrent or late onset manifestations of infection of the musculoskeletal or nervous system [6]. 

When there is a false-positive error (i.e., concluding that a rash is EM when in fact it is not), these individuals may seek out services unnecessarily, particularly if the observation from this study holds that the most common misidentification is for an uninfected benign tick bite. Of the two scenarios, not seeking help when help is needed versus seeking help when it is not needed, the latter is better for patients and public health as care is rendered. However, these individuals have sought care unnecessarily, incurring the cost of such services and potentially diverted resources away from individuals who could have received services sooner. 

This is the first study to use an internet-based survey in an attempt to capture the knowledge base of the general public (at least those interested enough in LD to seek out the website and take the survey) regarding the identifying of the most common sign of LD infection, the EM skin lesion. The internet has become a common way for the general public to collect information about signs and symptoms of diseases and the use of interactive health communication has risen due to increased use of internet and technology in health and research fields [29–31]. The benefits of interactive health communication include its convenience, ability to reach a larger slice of the population, and the ability to link individuals directly to health information. Using this interactive health communication tool, we were able to attract over 42,000 individuals to the website and entice over 3,900 individuals to participate in the survey in some form, with complete survey data from 3,104. Although we recognize that this is a relatively small sample given the number of individuals at risk for contracting LD, it provides a preliminary understanding of the target population's possible knowledge of the most common sign of LD. 

As this was a sample of convenience, we did not collect demographic information, their level of interest in LD, or information about their background training or education about LD. This population might have an above average interest and previous experience than the average non-medically trained individual. Without demographic information, it is hard to determine what, if any, specific group of the population would potentially benefit from an education intervention, or where geographically more education should be targeted. We believe that the individuals recruited for this survey are mostly nonhealth professionals. The website used for recruitment is not a health professional's reference site, nor is it linked to any reference site for health professionals. As stated previously, most of the traffic to the website resulted from searches on a search engine. Given this knowledge, even if the sample included health professionals with a higher level of knowledge about EM, the results would be overestimating the public's level of knowledge about LD rashes. 

With that said, the study findings support that there needs to be increased efforts to educate the general public, particularly individuals in endemic areas of the country about the multiple manifestations of the EM. As research continues, it is recommended that future studies include methods for collecting demographic information, including geographic location of respondents. This would allow geographical analyses to be done to see if those in Lyme endemic areas are more or less aware of the different manifestations of EM. It would also be important to include a question associated with each rash about the likelihood that the individual is likely to seek out consultation with health care professionals if he/she develops the particular rash. This would allow for greater understanding of the relationship between patterns of identification (correct, not correct, and undecided) with likelihood of seeking medical care. 

## 4. Conclusions

With increased knowledge of the general public's expertise in correctly identifying EM and seeking out services for medical care, public health education initiatives can be appropriately developed and targeted to address the deficiencies. While this survey did not target health care professionals, there is some evidence to suggest that physicians are limited in their ability to correctly identify the EM accompanying LD [14]. Another area for future research would be further investigating health provider's ability to accurately identify EM. Based upon those findings, training could be developed for front-line providers in Lyme endemic areas aimed at helping them to become familiar with the different appearances of EM. Such research could also provide the basis for guiding national agencies, such as the Centers for Disease Control, about the information they disseminate. 

Surveys such as the one reported here can be an important and influential public health tool. By assessing the gaps in knowledge of the public about a physical manifestation of LD, public health officials can target educational and outreach materials to these individuals. There is also opportunity for intervention following these types of assessments, such as education geared towards those in Lyme endemic areas and health care providers.
